# Enhancement and Limitations of Green-Spectrum Dual-Wavelength Irradiation in Porphyrin-Based Antimicrobial Strategies Targeting *Cutibacterium acnes* subsp. *elongatum*

**DOI:** 10.3390/pharmaceutics18010072

**Published:** 2026-01-05

**Authors:** Robin Haag, Oksana Gurow, Moritz Mack, Jörg Moisel, Martin Hessling

**Affiliations:** 1 Institute of Medical Engineering and Mechatronics, Ulm University of Applied Sciences, Albert-Einstein-Allee 55, 89081 Ulm, Germanymartin.hessling@thu.de (M.H.); 2 Medical Faculty of Ulm University, Meyerhofstraße 28, 89081 Ulm, Germany

**Keywords:** synergy, PDT, photosensitizer, CP III, visible light, ROS

## Abstract

**Background:** Phototherapy utilizes targeted irradiation to inactivate bacteria or treat various medical conditions. Depending on the therapeutic goal, wavelengths from violet to infrared (IR) are applied. Within the visible and near-IR spectrum, photodynamic therapy (PDT) combines light with photosensitizers that generate reactive oxygen species (ROS), leading to bacterial inactivation. Optimizing photodynamic efficacy can involve either enhancing ROS formation through specific topical agents that modulate ROS generation or employing dual-wavelength light irradiation (DWLR) to achieve synergistic excitation. Established DWLR protocols typically combine blue and red light or IR to activate distinct photosensitizers. **Materials and Methods:** This study investigates whether a similar synergistic effect can be achieved within the green spectral range by simultaneously exciting a single photosensitizer—coproporphyrin III (CP III)—at 496 nm and 547 nm. **Results:** Convolution analysis and in vitro bacterial reduction experiments with *Cutibacterium acnes* subsp. *elongatum* revealed that cyan irradiation (496 nm) achieved the strongest photoreduction (2.31 log steps at 1620 J/cm^2^), whereas PC-lime irradiation (547 nm) produced a smaller effect (0.74 log steps). DWLR protocols (simultaneous and sequential irradiation) resulted in intermediate reductions (1.64 and 1.73 log steps, respectively), exceeding PC-lime but not surpassing cyan irradiation alone. **Conclusions:** These findings demonstrate that excitation efficiency at the local absorption maximum of CP III is the primary determinant of ROS generation, while spectral broadening through DWLR does not enhance bacterial inactivation within this wavelength range and in vitro setup.

## 1. Introduction

In phototherapy, the application of light to treat various conditions has increased in clinical relevance. Treatments like photodynamic therapy (PDT) and intense pulse light therapy are established methods for mostly skin-related conditions like acne vulgaris, psoriasis, rosacea, viral warts, age-related changes, and even scars [1]. These therapies activate photosensitizers, which absorb light and generate reactive oxygen species (ROS), including superoxide radicals ($O_{2}^{∙-}$), hydroxyl radicals (HO-), and hydrogen peroxide (H_2_O_2_), as well as singlet oxygen (^1^O_2_), a non-radical excited state of molecular oxygen. The resulting highly reactive species damage nearby cellular components, leading to inactivation or cell death. To optimize the therapeutic outcome, topical agents are applied before irradiation. These include precursors such as aminolevulinic acid (ALA) and methyl aminolevulinate (MAL), which are metabolically converted into the photosensitizers coproporphyrin III (CP III) and protoporphyrin IX (PP IX), as well as directly applied photosensitizers such as indole-3-acetic acid (IAA) and methylene blue (MB) [2,3,4,5].

Photosensitizers absorb photons at specific wavelengths, promoting electrons from the ground state (S_0_) to excited singlet states (S_1_ and S_2_) via the HOMO–LUMO principle. From S_2_, rapid internal conversion leads to relaxation into S_1_, which then returns to S_0_ by fluorescence or undergoes intersystem crossing to a long-lived triplet state (T_1_). The triplet state is critical for ROS generation via two pathways: Type I (electron/hydrogen transfer with substrates, producing radicals that react with oxygen) and Type II (direct energy transfer to ground-state oxygen, producing singlet oxygen). Both pathways generate oxidative stress and inactivate microbial cells [1,6].

Photoinactivation efficiency can be enhanced by utilizing precursors, supplementing with additional porphyrins, or optimizing the irradiation spectrum to match porphyrin absorption. However, excessively high irradiation intensity induces photobleaching in which porphyrins become overexcited and structurally degraded, losing their ROS generating capacity. This degradation compromises inactivation efficacy and may allow microorganism survival due to diminished absorption and ROS formation [7,8]. Therefore, dual-wavelength light irradiation (DWLR) uses two wavelengths (applied sequentially or simultaneously) to excite light-sensitive molecules and engage multiple inactivation pathways [9,10,11]. Sequential DWLR has revealed benefits in MRSA (methicillin-resistant *Staphylococcus aureus*), where 460 nm pre-irradiation photo-oxidizes the protective pigment staphyloxanthin (STX) and sensitizes bacteria to subsequent 405 nm light [11], and in acne vulgaris, where 1450 nm laser treatment targeting sebaceous activity followed by 450 nm blue light yields greater lesion reduction than blue light alone [10]. Boonpethkaew et al. found similar synergy with 589 nm red light followed by 1319 nm irradiation [12]. Through simultaneous DWLR combining hydrogen peroxide with toluidine blue O or indocyanine green and irradiation at 980 nm + 635 nm or 980 nm + 808 nm, stronger antimicrobial effects and higher ROS levels are produced compared to either PDT or hydrogen peroxide alone [9]. These findings indicate that DWLR can produce more than additive effects, i.e., true synergy.

Synergy in photodynamic therapy refers to a therapeutic effect that exceeds the simple additive action of the individual components. In DWLR protocols, this means that combined irradiation with two wavelengths achieves higher efficacy than the sum of each wavelength alone because complementary mechanisms, such as protein damage, impaired DNA repair (I), direct and indirect DNA damage (II), and ROS generation via endogenous photosensitizers (III), are activated in parallel. As summarized by Matafonova et al., synergy occurs when two spectral ranges are combined (I + II, I + III, or II + III), but certain wavelength pairs fail to show synergy, so DWLR is not universally synergistic, and most evidence to date comes from water disinfection studies using UV radiation [13]. Human and skin-targeted therapies predominantly employ wavelengths in the visible to near-infrared range [14]. In human DWLR PDT, direct DNA damage is unlikely because UV wavelengths are generally avoided for safety reasons. The most relevant mechanisms are (a) photosensitizer activation at distinct absorption peaks, broadening and enhancing ROS generation; (b) photodamage of protective pigments or defense structures (e.g., STX in *Staphylococcus aureus*), reducing microbial resistance; and (c) complementary penetration depths, enabling simultaneous treatment of superficial and deeper targets. Together, these mechanisms explain how DWLR PDT can exceed additive effects and achieve genuine synergy [11,13,14].

This introduction focuses on antimicrobial photodynamic therapy (aPDT), but the underlying concepts and mechanisms also apply to eukaryotic cells. PDT, including DWLR, is long established in oncology and has also been studied in plant biology, where light stress on leaf tissues illustrates wavelength-dependent damage [15,16,17,18,19]. Thus, light exposure can affect prokaryotic cells such as *Cutibacterium acnes* (*C. acnes*) and other bacteria, as well as human skin and tissue, depending on wavelength, intensity, and treatment conditions. According to the European Dermatology Forum guidelines on topical PDT (2019, Part 2), aPDT for acne vulgaris is considered safe and supported by substantial clinical evidence, with typically mild, transient adverse effects and no long-term or photocarcinogenic risks identified. Compared with oncologic PDT, dermatologic applications such as acne therapy use lower photosensitizer doses and smaller treatment fields and are generally less limited by treatment-associated pain [20].

Sequential DWLR in acne has shown synergistic clinical effects, as mentioned above, but does not consistently produce a synergistic outcome. In contrast, Ryu et al. used an intense pulsed light device with a dual-band filter (400–600 nm and 800–1200 nm) to deliver visible and IR spectra simultaneously; although no synergy was demonstrated, the treatment was still effective for acne vulgaris [21]. Two additional clinical studies using sequential blue (415 nm or 470 nm) and red (633 nm or 640 nm) light reported better therapeutic outcomes than single-wavelength regimens, but the authors did not explicitly describe synergistic effects [22,23].

This study explores the potential synergistic effects of DWLR in the green spectral range, specifically at 496 nm and 547 nm, on *Cutibacterium acnes* subsp. *elongatum*. *C. acnes* is a natural member of the human skin microbiome. Disruption or imbalance of this microbial community, particularly involving *C. acnes*, has been linked to the pathogenesis of acne vulgaris. Clinically, acne vulgaris is most commonly associated with phylotype I strains of *C. acnes*, while phylotype III strains, such as *C. acnes* subsp. *elongatum*, are not typically implicated in inflammatory skin conditions. Nevertheless, *C. acnes* subsp. *elongatum* belongs to Risk Group 1 and can therefore be safely employed under standard laboratory conditions in contrast to phylotypes I and II, which are classified as Risk Group 2 [24,25]. Although not directly pathogenic, *C. acnes* subsp. *elongatum* shares close genetic similarities with acne-associated phylotypes and represents a natural skin commensal, making it a suitable organism for investigating DWLR effects.

Wavelengths of 496 nm and 547 nm were chosen according to the absorption profile of CP III, the predominant porphyrin produced by *C. acnes* [26]; within the green spectrum, they coincide with two Q band absorption maxima. CP III absorbs most strongly in the Soret band around 405 nm, but this blue light region is heavily attenuated in skin, leading to shallow penetration and mainly superficial effects, whereas green light penetrates more deeply and is therefore advantageous for aPDT despite lower CP III absorption and ROS yield at these wavelengths. Targeting the Q bands in the green range represents a compromise between tissue penetration and porphyrin activation.

Moreover, applying both wavelengths together, either simultaneously or sequentially/alternating, may enhance the overall outcome by covering multiple absorption peaks, thereby enabling potential synergistic effects. Importantly, such comparisons must be performed at constant fluence to evaluate possible synergy. To ensure a consistent and controlled environment, *C. acnes* subsp. *elongatum* was irradiated in planktonic state and under a laboratory setup without any other influence except the radiation.

## 2. Materials and Methods

### 2.1. Microbiology

In this research, *Cutibacterium acnes* subsp. *elongatum* (JCM 18919) from the Japanese Collection of Microorganisms was investigated. The bacterium was cultured using Brain Heart Infusion (BHI) for agar plates and liquid medium. For experimental procedures, cryopreserved *C. acnes* subsp. *elongatum* stocks were streaked onto BHI agar plates and incubated anaerobically at 37 °C for five to seven days. Anaerobic conditions were maintained using ‘Anaerocult C mini^®^’ pads from Merck (Darmstadt, Germany), enclosed in sealed plastic bags. After incubation, selected bacterial colonies were carefully harvested with an inoculation loop and suspended in phosphate-buffered saline (PBS, pH 7.3). The bacterial concentration was determined by measuring the Optical Density @ 600 nm (OD_600_) with a Specord 250 Plus spectrometer (Analytic Jena, Jena, Germany). The suspension was adjusted to an OD_600_ value of 0.03, corresponding to an estimated 10^7^ colony-forming units per milliliter (CFU/mL). Next, four samples of 3 milliliters of the prepared bacterial suspension were transferred into small beakers and placed within the irradiation setups. These four samples were irradiated simultaneously with different modes. An additional 3-milliliter sample was used as a control. Samples were collected at specific time points—0 h, 3 h, 6 h, and 9 h—and subsequently plated onto BHI agar plates for incubation under anaerobic conditions at 37 °C for seven days. Throughout the irradiation procedure, temperature and sample weight were monitored. In cases of measurable evaporation, sterile distilled water was added to compensate for any loss. Coproporphyrin III (CP III; CAS: 14643-66-4; Santa Cruz Biotechnologies, Dallas, TX, USA) was dissolved by preparing a stock solution containing 2.6 mg of CP III in 100 µL of dimethyl sulfoxide (DMSO; CAS: 67-68-5; SERVA Electrophoresis GmbH, Heidelberg, Germany). For absorbance measurements, 1 µL of the stock solution was diluted in 999 µL phosphate-buffered saline (PBS). Spectral analysis was conducted using μClear^®^ 64-well half-area microplates (Greiner Bio-One, Frickenhausen, Germany) and a CLARIOstar^®^ microplate reader (BMG Labtech, Ortenberg, Germany). Each well was filled with 50 µL of the diluted sample, and absorbance was recorded across a wavelength range of 350 to 800 nm. Measurements were performed in triplicate.

### 2.2. Setup

**Figure 1:**
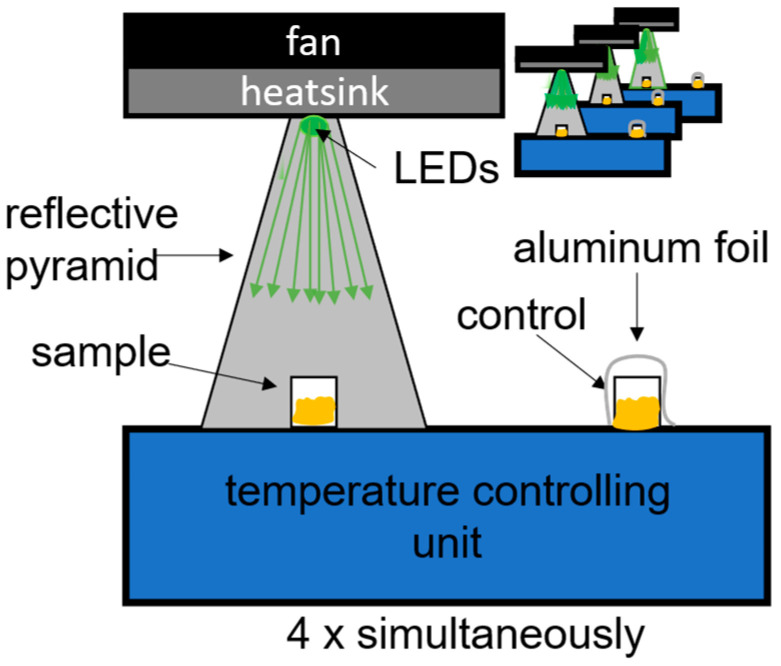
Schematic representation of the experimental setup. From top to bottom: LED with cooling system on top; reflective pyramid; irradiated sample positioned inside the pyramid; and a control sample, shielded with aluminum foil to prevent exposure to light.

The irradiation system was constructed around a “Thermocell Cooling and Heating Block” (Bioer; Model: CHB-202; Hangzhou, China), thereby ensuring a stable temperature of 35 °C throughout the experiment. A custom-fabricated 3D-printed holder securely positioned both the irradiated sample and the control. To optimize the light pathway, a reflective pyramid directed illumination from the LEDs to the sample, ensuring more homogeneous irradiation. The LED module mounted at the top of the setup incorporated a cooling mechanism consisting of a heatsink and a fan to maintain thermal stability. For a detailed schematic, refer to Figure 1. This setup is similar to setups used by McKenzie et al. and Hoenes et al. in investigating planktonic bacteria [27,28].

### 2.3. LEDs and Irradiation Settings

In this experiment, the following two LEDs from the XE-G series form Cree^®^ (Durham, NC, USA) were used: cyan (XEGACY-H-C20-T10-F000-000-0001) and PC-lime (phosphor-converted lime; XEGAPL-H-PL4-V30-0000-000-0001). The LEDs were measured using an integrating sphere and a CAS140D type spectrometer from Instrument Systems (Munich, Germany). The four different irradiation setups are presented in Table 1 below.

### 2.4. Evaluation, Statistics, and Mathematical Illustration

The evaluation of the CFU on agar plates was performed with the SphereFlash^®^ (IUL; Barcelona, Spain), a device which counts developed colonies automatically. Then, CFU data were further processed with Excel^®^ (Microsoft; Redmond, WA, USA). The CP III absorptivity spectrum and the irradiation spectra were also documented in Excel. In addition, the spectrum of CP III absorptivity and the different irradiation spectra became convoluted, as demonstrated in Equation (1). This resulted in four different spectra due to four irradiation protocols (see Table 1). The fluence from the simultaneous irradiation and the single-wavelength irradiation was directly measurable, and the alternating spectrum was determined as the mean of the convolution of the cyan and the PC-lime irradiation, leading to the four different results mentioned. The area under the curve (AUC) of the resulting function was calculated as presented in Equation (2). These values were combined with the absolute gradient of the log-reduction fit-function and displayed in a shared diagram. Additionally, as classical photochemical quantum yields are defined as the number of events per absorbed photon, this work presents a photobactericidal efficacy parameter E_PB_(λ) defining the ratio between absorbed photon and caused reduction event. The photobactericidal efficacy parameter E_PB_(λ) is determined as presented in Equation (3) and graphically displayed next to the AUC results.

(1) $$C\left(\lambda \right)={(1-10}^{-A\left(\lambda \right)})\times E_{LED}(\lambda )$$

C(*λ*) denotes the convolution spectrum obtained as the product of the CP III absorptivity spectrum A(*λ*) and the irradiation emission spectrum E_LED_(*λ*). Since four different irradiation protocols were applied, *E*_LED_(*λ*) corresponds to four distinct emission spectra, each resulting in a separate convolution spectrum C(*λ*).

(2) $$AUC=\int_{\lambda =400\;nm}^{700\;nm}C(\lambda )d\lambda$$

This describes the calculation of the area under the curve (AUC) of the newly created ‘spectrum’ C(*λ*) in Equation (1). The integration boundaries *λ*_min_ = 400 nm and *λ*_max_ = 700 nm are determined to consider all aspects of the convolution C(*λ*).

(3) $$E_{PB}\left(\lambda \right)=\frac{\log reduction\left(\lambda \right)}{\frac{LED\;power\;\times \;exposure\;time\;\times \;wavelength}{h\;\times \;c}\times \left(1-10^{-A\left(\lambda \right)}\right)}\left[\frac{\log reduction}{photon}\right]$$

This defines the photobactericidal efficacy E_PB_(λ) expressed in units of log reduction per absorbed photon. The log reduction at wavelength λ is derived from the inactivation data. The incident fluence (LED power × exposure time) for all irradiations was 1620 J/cm^2^. The parameter wavelength is determined by the irradiation wavelength. The constants used are Planck’s constant (h = 6.62 × 10^−34^ Js) and the speed of light (c = 299,792,458 m/s). As the absorptivity factor, the average CP III absorptivity over the respective irradiation spectrum is used.

## 3. Results

### 3.1. Spectra

**Figure 2:**
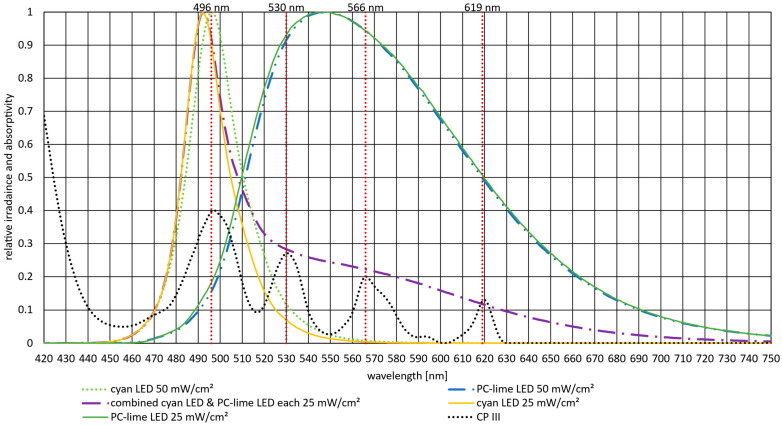
Absorptivity spectrum of CP III alongside the normalized emission spectra of the cyan and PC-lime LEDs measured at two irradiances (25 mW/cm^2^ and 50 mW/cm^2^). The vertical red lines indicate the local absorption maxima of CP III within the Q band, with the numerical labels referring to the exact peak wavelengths. Slight variations in the LED emission spectra originate from changes in electrical current causing a minor red shift (and the two different irradiances). The cyan LED at 50 mW/cm^2^ has an emission peak at 496 nm and a slightly shifted peak of 493 nm at 25 mW/cm^2^. The emission peak of PC-lime is consistently observed at 547 nm.

Figure 2 presents the emission spectra of the cyan and PC-lime LEDs used for irradiation alongside the absorption spectrum of CP III. The vertical red dotted lines indicate the local absorption maxima of CP III in the Q band at 496 nm, 530 nm, 566 nm, and 619 nm. Each LED spectrum was measured at two irradiances, 25 mW/cm^2^ and 50 mW/cm^2^. Minimal differences were observed between the spectra, likely attributable to variations in current, which caused this minor red shift in the emission peaks. For the experiments, the lower irradiance of 25 mW/cm^2^ was applied simultaneously to match the single irradiance of 50 mW/cm^2^. Nevertheless, the spectra were measured separately and then combined to illustrate the origin of the shifts observed in the combined spectrum compared with the 50 mW/cm^2^ irradiance measurements. Notably, the emission spectra of both LEDs exhibit considerable overlap with the Q band absorption maxima of CP III, indicating that both sources are spectrally well suited for excitation.

### 3.2. Reduction in C. acnes subsp. elongatum by Irradiation Within the Green Spectra

**Figure 3:**
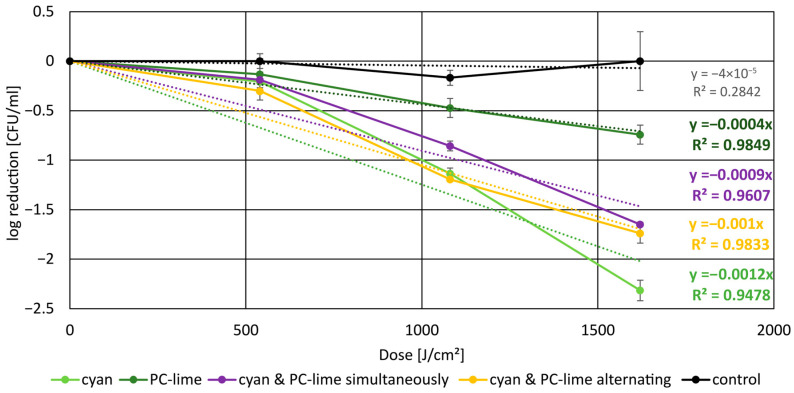
Reduction in *C. acnes* subsp. *elongatum* under different irradiation conditions, including linear fit functions. The black line represents the unirradiated control group, which maintained a nearly constant bacterial concentration. At a fluence of 1620 J/cm^2^, PC-lime irradiation at 50 mW/cm^2^ for 9 h (dark green line) resulted in a reduction of 0.74 log steps, while simultaneous irradiation with cyan and PC-lime at 25 mW/cm^2^ each (violet line) achieved 1.64 log steps, and alternating irradiation (yellow line) yielded 1.73 log steps. The greatest reduction was obtained with cyan irradiation alone, reaching 2.31 log steps (green line).

The inactivation kinetics of *C. acnes* subsp. *elongatum* under different irradiation regimens are summarized in Figure 3. All irradiated samples exhibited a characteristic shoulder effect at the beginning of the irradiation, consistent with an adaptation or repair capacity before effective photoinactivation dominated. At equal total fluence (1620 J/cm^2^), distinct differences in efficacy were observed between the single wavelength and DWLR approaches. While PC-lime irradiation alone produced only modest reductions, the combination of cyan and PC-lime—whether applied simultaneously or sequentially—resulted in substantially greater bacterial inactivation. The most pronounced effect was observed with cyan irradiation alone with 2.31 log steps in reduction, likely due to higher absorption with the Q band absorption maxima of CP III, leading to more efficient porphyrin activation. From the perspective of PC-lime irradiation, combining PC-lime with cyan in a DWLR approach resulted in a synergistic reduction effect at the same total fluence, clearly surpassing PC-lime alone. However, when viewed in comparison to cyan irradiation, no additional benefit was observed, indicating that the dual-wavelength approach does not exceed the efficacy achieved with cyan light itself.

### 3.3. Convolution Analysis, Gradient Comparison, and Photobactericidal Efficacy

**Figure 4:**
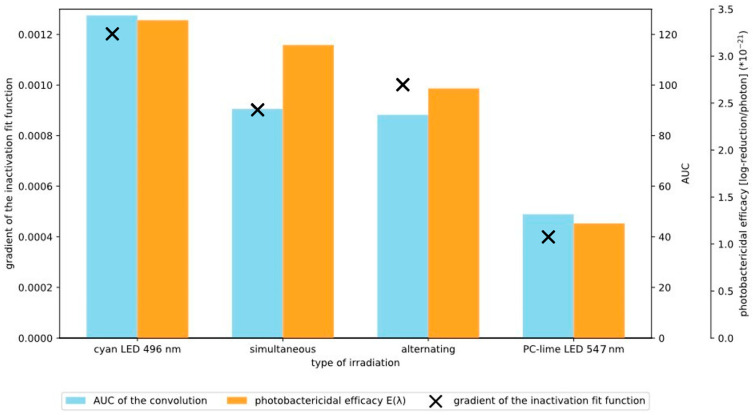
Quantitative comparison of ‘spectral overlap’, bacterial inactivation, and photobactericidal efficacy. Blue bars represent the area under the curve (AUC) of the convolution between the CP III absorption spectrum and the respective LED emission spectra, serving as an estimate of excitation efficiency of CP III creating ROS. The orange bars represent the photobactericidal efficacy E_PB_ for every irradiation type (the scale of the corresponding axis on the right is ×10^−21^). Black crosses indicate the corresponding inactivation gradients derived from the reduction kinetics (see Figure 4). This figure enables direct comparison between the spectra applied and the biological reduction effect. The values displayed for the AUC calculation are as follows from left to right: 127, 90, 88, and 78. The values for the photobactericidal efficacy E_PB_ from left to right are 3.38 × 10^−21^, 3.11 × 10^−21^, 2.65 × 10^−21^, and 1.21 × 10^−21^.

To further quantify the irradiation outcomes, the AUC of the convoluted spectra (blue bars) was paired with their matching reduction gradient (‘X’) and photobactericidal efficacy (orange bars) in Figure 4. Convolution of the CP III absorptivity spectrum with the respective LED emission spectrum provides an estimate of effective excitation and, consequently, the potential ROS production efficiency. These graphically illustrated results resemble a similar trend across the three metrics, as shown in Figure 4. This analysis illustrates that both the simultaneous and sequential DWLR produced a larger AUC compared to PC-lime irradiation alone, indicating a more effective inactivation over time. Interestingly, the gradient of the reduction curves for the DWLR approaches more closely resemble that of cyan irradiation, suggesting that the overall inactivation dynamics were dominated by cyan absorption. This observation aligns with the strong spectral overlap of cyan emission with the Q band maxima of CP III, resulting in efficient porphyrin excitation. Consequently, while DWLR provides a clear synergistic benefit relative to PC-lime alone, it does not exceed the efficacy of cyan irradiation when considered in isolation. Figure 4 also provides insights into the relationship between the spectra applied and the caused bacterial reduction.

## 4. Discussion

This study investigates the capacity of light-based reduction within the green spectral range by comparing single-wavelength irradiation to DWLR, with the aim of identifying potential synergistic effects arising from simultaneous excitation of the same porphyrin molecule. The corresponding spectral data were quantitatively analyzed using convolution to reveal how the spectral match between the irradiation and porphyrin absorption influences photodynamic efficacy. The results demonstrate that cyan irradiation alone has the highest level of photoinactivation, underscoring the dominant role of spectral emission overlap with the Q band maxima of CP III. This finding is further supported by the convolution-based AUC analysis in which calculated values closely reflected the experimentally observed inactivation kinetics. Notably, the photobactericidal efficacy was further quantified and found to be in close agreement with the AUC-based spectral analysis, demonstrating that wavelength-selective excitation efficiency can be directly predicted from the overlap integral between LED emission spectra and the CP III absorption profile. This quantitative correspondence between spectral convolution and experimental inactivation kinetics establishes a mechanistic link between photophysical parameters and antimicrobial outcome, enabling rational optimization of light sources for photodynamic applications against *C. acnes* subsp. *elongatum*. Hence, within the context of this study, excitation efficiency at the relevant porphyrin absorption peaks constitutes a more decisive factor for antimicrobial efficacy than spectral broadening alone achieved by DWLR exposure. Previous studies have investigated the photodynamic efficacy of light against *C. acnes* mostly in clinical ways, as mentioned in the Introduction. This work is an approach to correlate spectral characteristics with antimicrobial outcome and therefore addresses the need for a quantitative, mechanistic understanding of the underlying photophysical mechanisms that govern how wavelength selectivity and spectral overlap with endogenous porphyrin absorption directly influence photodynamic inactivation efficiency.

When compared to the recent literature, these results highlight nuanced parallels and limitations. Some studies, as mentioned in the Introduction, have shown synergistic in vivo outcomes when combining wavelengths that target distinct mechanisms—such as blue and (infra-) red light in combination, which can activate different cellular pathways and penetrate tissue at varying depths [10,12]. As another example, Astuti et al. investigated bacterial reduction and wound healing in mice using blue (392 nm) and red (627 nm) LEDs, leveraging separate absorption peaks for increased photodynamic action [29]. Notably, in this study, the authors differentiate the effectiveness of infected wound healing in an in vivo setup including mice and in vitro bacterial inactivation of *Staphylococcus aureus*. They conclude that infectious wounds heal better with both LEDs, whereas *S. aureus* is inactivated best by blue LED alone [29]. Similarly, Leanse et al. demonstrated enhanced killing of MRSA through sequential 460 nm and 405 nm irradiation, where pre-excitation actively disrupts protective pigments, amplifying the subsequent effect [11]. In contrast to this study, it focused on dual wavelengths within the same region, primarily acting via the same porphyrin system.

Several study limitations warrant consideration. The use of the Risk Group 1, non-inflammatory *C. acnes* subsp. *elongatum* ensures laboratory safety but limits direct clinical extrapolation to acne-associated phylotypes [30]. In vitro conditions may not fully replicate the complexity of in vivo environments, particularly regarding oxygen gradients, tissue penetration, and homogeneous irradiation. If these findings are extrapolated to a clinical setting, it is important to note that while cyan light offers superior porphyrin activation, its limited tissue penetration constrains clinical effectiveness. In contrast, PC-lime and longer wavelengths penetrate more deeply but are less efficient at exciting porphyrins for ROS production. This presents a trade-off between excitation efficiency and tissue depth [31]. Furthermore, the literature reporting synergistic effects in DWLR most often utilizes combinations of different mechanisms, molecules, or agents to enhance bacterial reduction. There was no prior study that addressed the optimization of ROS generation from a single photosensitizer molecule under DWLR. In addition, future studies in this context would benefit from considering surrounding biological factors such as skin penetration depth and the potential for photobleaching. This reveals the need for further investigation into wavelength-dependent photochemistry that seeks to maximize microbial inactivation without compromising photosensitizer integrity, particularly for clinical translation where tissue optics and selective targeting significantly impact therapeutic outcomes.

Overall, these findings advance the mechanistic understanding of how spectral design influences the efficacy of DWLR in an in vitro setting. Convolution analysis has been identified as a potentially valuable predictive tool for the optimization of light-photosensitizer combinations, thereby facilitating the prioritization of wavelengths based on their local absorption maximum overlap. Concurrently, dual-wavelength methodologies maintain their relevance in clinical applications by potentially balancing the absorption efficiency with the tissue penetration depth and the ROS generating capacity. This study does not make any medical application suggestions, because the extrapolation of in vitro findings is not an indicator of in vivo success because of additional complex factors. Astuti et al. observed greater reduction with single irradiation in vivo but better healing of infected wounds in mice with DWLR [29].

## 5. Conclusions

This study investigated the potential synergistic effects of dual-wavelength irradiation (DWLR) for activation of ROS in CP III. In vitro experiments and convolution analysis of relevant spectra identified 496 nm (cyan) irradiation as the most effective wavelength for bacterial reduction, achieving a maximum decrease of 2.31 log steps at a fluence of 1620 J/cm^2^. Irradiation at 547 nm (PC-lime) produced a lesser reduction of 0.74 log steps under the same conditions. DWLR protocols resulted in enhanced bacterial reduction compared to PC-lime irradiation alone, with simultaneous and alternating regimens achieving reductions of 1.64 and 1.73 log steps, respectively. However, the combined approaches did not exceed the efficacy observed with cyan irradiation alone. These findings indicate that targeting the local absorption maximum of the photosensitizer is more critical for efficient ROS production than broadening the excitation spectrum or incorporating irradiation at wavelengths corresponding to lesser absorption peaks in this in vitro setting.

## Figures and Tables

**Table 1:** Summary of the four irradiation setups used in the experiment. The setups include continuous irradiation with either cyan or PC-lime at 50 mW/cm^2^, simultaneous dual-wavelength irradiation at 25 mW/cm^2^ each, and alternating irradiation every 5 min with 50 mW/cm^2^ per wavelength.

Setup	Active LED	Irradiance [mW/cm^2^]	Irradiation Mode
1	cyan	50	continuous
2	PC-lime	50	continuous
3	cyan + PC-lime	25 + 25	simultaneous
4	cyan ⇄ PC-lime	50 ⇄ 50	alternating every 5 min

## Data Availability

All data supporting the findings of this study are included within the article. Additional data is available from the corresponding author upon reasonable request.
